# Tips for permanent education in mental health in primary care guided by the Institutional Socio-clinic*

**DOI:** 10.1590/1518-8345.3217.3204

**Published:** 2019-10-28

**Authors:** Larissa de Almeida Rézio, Cinira Magali Fortuna, Flávio Adriano Borges

**Affiliations:** 1 Universidade Federal do Mato Grosso, Faculdade de Enfermagem, Cuiabá, MT, Brazil.; 2 Scholarship holder at the Coordenação de Aperfeiçoamento de Pessoal de Nível Superior (CAPES), Brazil, and at the Fundação de Amparo à Pesquisa do Estado de Mato Grosso (FAPEMAT), Brazil.; 3 Universidade de São Paulo, Escola de Enfermagem de Ribeirão Preto, PAHO/WHO Collaborating Centre for Nursing Research Development, Ribeirão Preto, SP, Brazil.; 4 Universidade Federal de São Carlos, Departamento de Enfermagem, São Carlos, SP, Brazil.

**Keywords:** Permanent Education, Professional Training, Public Health, Mental Health, Primary Health Care, Nursing in Public Health, Educação Permanente, Formação Profissional, Saúde Pública, Saúde Mental, Atenção Primária à Saúde, Enfermagem em Saúde Pública, Educación Continua, Capacitación Profesional, Salud Pública, Salud Mental, Atención Primaria de Salud, Enfermería em Salud Publica

## Abstract

**Objective:**

to analyze a process of Permanent Education in Health about mental health with Family Health teams.

**Method:**

research-intervention performed with 20 workers from two teams of the Family Health Strategies. Semi-structured interviews and 12 reflection meetings were carried out with each team. The principles of Institutional Socio-clinic were used to guide the meetings and the analysis of the data.

**Results:**

seven beaconing tips were identified for the Process of Permanent Education in Health: effects produced from the choices of inclusion of the management in the planning of the meetings, revealing established ways of working; attention to non-control in training movements; use of restitution at meetings, reducing stiffness and tensions; attention to the institutions that cross us; analysis of the facilitator’s involvement in the training, redirecting behaviors and attitudes; problematization about the object, instrument and purpose, which favored the reflection about the mental health care and to learn to facilitate and experience the Permanent Education in Health in the act of making.

**Conclusions:**

socio-clinic assisted the experience of facilitating in-service training, pointing out tips for the collective construction of contextualized, reflexive and problematizing knowledge.

## Abstract (pt)

**Objetivo:**

analisar um processo de Educação Permanente em Saúde sobre saúde mental com equipes de Saúde da Família.

**Método:**

pesquisa-intervenção realizada com 20 trabalhadores de duas equipes da Estratégias de Saúde da Família. Foram realizadas entrevistas semiestruturadas e 12 encontros de reflexão com cada equipe. Utilizaram-se os princípios da Socioclínica Institucional para guiar os encontros e a análise dos dados.

**Resultados:**

foram identificadas sete pistas balizadoras para o processo de Educação Permanente em Saúde: efeitos produzidos a partir das escolhas de inclusão da gestão no planejamento dos encontros, revelando modos instituídos de trabalho; atenção ao não controle nos movimentos de formação; utilização da restituição nos encontros, diminuindo o enrijecimento e tensões; atenção às instituições que nos atravessam; análise de implicação da facilitadora da formação, redirecionando comportamentos e atitudes; problematização acerca do objeto, instrumento e finalidade, que favoreceram a reflexão acerca do cuidado em saúde mental e aprender a facilitar e vivenciar a Educação Permanente em Saúde no ato de fazer.

**Conclusão:**

a Socioclínica auxiliou a vivência de facilitar a formação em serviço, apontando pistas para a construção coletiva de conhecimento contextualizado, reflexivo e problematizador.

## Abstract (es)

**Objetivo:**

analizar el proceso de Educación Permanente en Salud sobre salud mental con equipos de Salud de la Familia.

**Método:**

investigación intervención realizada con 20 trabajadores de dos equipos de la Estrategias de Salud de la Familia. Fueron realizadas entrevistas semiestructuradas y 12 encuentros de reflexión, con cada equipo. Se utilizaron los principios de la Clínica Social para guiar los encuentros y analizar los datos.

**Resultados:**

fueron identificados siete indicios señalizadores del proceso de Educación Permanente en Salud: efectos producidos a partir de las elecciones de inclusión de la administración en la planificación de los encuentros, revelando modos instituidos de trabajo; dar atención al no control, en los movimientos de formación; utilización de la restitución en los encuentros, disminuyendo la rigidez y tensiones; dar atención a las instituciones que nos atraviesan; analizar la implicación de la facilitadora de formación, redirigiendo comportamientos y actitudes; problematizar sobre el objeto, instrumento y finalidad, que favorecerán la reflexión sobre el cuidado en salud mental; y, aprender a facilitar y experimentar la Educación Permanente en Salud en el acto de hacer.

**Conclusiones:**

la Clínica Social auxilió la vivencia de facilitar la formación en el trabajo, apuntando indicios para la construcción colectiva del conocimiento contextualizado, reflexivo y problematizador.

## Introduction

The National Health Council (NHC) approved the Training and Development Policy for the Unified Health System (UHS): Pathways to Permanent Education in Health, establishing the National Permanent Education Policy (NPEP) as a UHS strategy for training and work development, considering the constitutional responsibility of the Ministry of Health to order the formation of human resources for the health area^(^ 1 ^)^.

It is understood that the Permanent Education in Health (PEH) is a proposal of relevant learning to contemplate the worker as protagonist of the formation process. The latter - also focused on the problems and difficulties experienced in the daily production of care, management and participation and social control - makes it possible to construct collective spaces for reflection and evaluation of the daily actions of the health services, decentralizing and disseminating the pedagogical capacity between managers and workers, operating in the micro-politics of the work process. In this way, professional training is offered at the same time as the changes in health practices are produced^(^ 2 ^-^ 4 ^)^.

PEH is one of the strategies that can favor the expansion of mental health care in relation to health services and the care network, as well as highlighting various possibilities of work tools to be used in the psychosocial context, since new policies and guidelines guide the care network and, therefore, involve workers from different services and, necessarily, the Family Health Strategy (FHS), seeking the construction of autonomy and social reintegration of the subjects^(^ 5 ^-^ 6 ^)^.

Despite this indicative of network care, studies indicate that the FHS teams report difficulties in providing care to people suffering from mental illness, referring to the fragility of this specific care and the need for training for it^(^ 7 ^-^ 11 ^)^.

In addition, it is relevant that Mental Health care is also understood in the context of general health, in which the worker acknowledges that the mental health demand can be present in several complaints reported by the users that reach the FHS. Thus, thinking about PEH as a strategy capable of generating reflective processes and covering mental health care can result in comprehensive care of the person and the family.

Given this context, there is a new demand for the training of workers at work, for work and for the work ^(^ 12 ^)^. In order to expand understanding and look at mental health as a care integrated with practice and that takes into account the integrality of the subject, it is necessary that the training be based on the reflection of the work process and that it is a collective construction, not taxation and encompasses psychosocial attention as a guideline.

Although there is this need for training, most of the international studies point to a formative logic still focused on the traditional training and capacities, with measurements of their impacts in the daily work^(^ 13 ^-^ 17 ^)^.

Another relevant point refers to the fact that Mental Health training is often based on the biomedical / psychiatric model, emphasizing the diagnosis of mental disorders and drug treatments, besides happening in a decontextualized way of the daily work of health^(^ 18 ^)^.

Brazil has stood out in terms of training that takes place from the context of health work, dialoguing with it, in a permanent perspective, seeking to propitiate reflection, problematizations, denaturalization of concepts and hegemonic practices in Mental Health, in addition to discussions on the different perspectives regarding psychic suffering and psychosocial attention, incorporation of the discussion of interventions present in the daily routine of the practice as the reception and qualified listening, expanding the ways of caring for the health of the population^(^ 19 ^-^ 20 ^)^.

Given this context, it is understood that the training is not effective to enable the change of practice and the incorporation of new concepts, by de-contextualization and based only on the transmission of knowledge in a unidirectional perspective^(^ 3 ^)^.

Like PEH, one of the structuring axes of Institutional Socio-clinic is also work. For this, the professional practice includes ways of relating to the collective of work, of thinking about these relations and of assigning values, being constituted by a set of updates of the relations that the subjects establish with the professional institution, that is, its professional implications^(^ 21 ^)^.

The institution, in turn, would be the rules and rules established and socially constructed, such as family, education, health, work, among others^(^ 22 ^)^. In this sense, the individual and / or collective subject can problematize his daily work, recognizing himself involved with the aspects and institutions that he questions.

It is believed, therefore, that the Institutional Socio-clinic can act in the perspective of generating PEH processes. It does not consist in a technical modality or protocol of practice and analysis, but in a way to question the object and intentions of analysis, seeking to understand the social dynamics^(^ 21 ^)^.

Its principles consist of characteristics to be observed in the beaconing of institutional interventions. They are: participation of the subjects in the device; analysis of order and demands; analyzer work; application of refund arrangements; analysis of the transformations that occur as the work progresses; intention of knowledge production; attention to contexts and institutional interference, and the analysis of the implications^(^ 21 ^)^.

The devices are elements such as writing, speech, videos, among others, created for / in situations of intervention, that can destabilize the instituted ways of functioning of the institutions, and can also become an analyzer if they can put some situation under analysis, revealing the structure of the institution, provoking it and forcing it to speak^(^ 21 ^,^ 23 ^-^ 24 ^)^.

The order refers to an official request for imaginary solutions or actions to restore order and allows the intervention to begin. It involves not only who asks for the intervention, but also what is asked for^(^ 23 ^)^. The demands are conscious, manifest, deliberate aspects and also unconscious and unsaid aspects, and may be in tune with the order or not. Thus, the order also has individual demands^(^ 23 ^,^ 25 ^)^.

During the analytical process, transformations occur that, for Socio-clinic, deserve attention and attention. In the same way, there are institutional interferences, processes of intersection and noise produced when institutional logics enter into contradiction.

In order for this process to take place, it is necessary to analyze the implications of the researcher and the other participants in the development of socio-clinical research. Analyzing the implications is to analyze the relationships that the subjects establish with the institutions that pass through them^(^ 26 ^)^.

Using the principles of Institutional Socio-clinic to mark PEH in Mental Health in the FHS can be a potentiator of learning processes and cause workers to think and reflect on their respective work processes.

Therefore, aiming to answer the research question of how Socio-clinic can enhance the development of PEH in Mental Health in the FHS this study aims to analyze a process of PEH in Mental Health with FHS teams, guided by the principles of Institutional Socio-clinic, pointing out tips to the development and construction of a critical, reflexive, contextualized and problematizing knowledge.

## Method

Research-intervention based on the theoretical reference of Institutional Analysis - Socio-clinic and carried out from March 2016 to February 2017. The intervention research has the political character of search for transformations from the interrogation of the various senses crystallized in institutions, broadening the theoretical bases and methodologies, in addition to enabling the production of knowledge and new practices at the same time. In this way, the researcher is the subject and object of knowledge^(^ 27 ^)^.

The data presented comprised part of a doctoral thesis whose field of intervention was composed by two FHS of a large municipality of the State of Mato Grosso.

All the workers from the participating FHS, interested in the PEH / Mental Health process, were invited to participate in the intervention research, totaling 20 subjects: eleven workers from team I and nine workers from team II, eleven community health agents (CHA); two doctors; two nurses; two nursing techniques; two receptionists and a typist. This number fluctuated during the training meetings so that each group had, on average, five to seven people participating.

As an instrument, the semi-structured interview with all workers was used as an instrument, seeking information about daily work, Mental Health practices in the FHS and experiences in PEH. Based on the answers, the analysis and the group reimbursement, themes were included to guide the training sessions in Mental Health. The topics studied were: organizational proposals based on NPEP; psychiatric reform and Mental Health network care; reception and approaches to the user in mental suffering and in use of psychoactive substance; team work; singular therapeutic design; use of tools / scales / tools for Mental Health care and Mental Health care strategies in the FHS. These training meetings were also configured as data production space.

The PEH was guided by the principles of Institutional Socio-clinic with the workers who accepted to participate, totaling 12 meetings, with an average of two hours. The meetings were recorded and transcribed for analysis, protecting the identity of participants.

One also used the researcher’s field diary on the intervention research. In this diary, there were reports of group movements, resistances, advances; content discussed, that is, the narrative of the historical-social context reflected in the research activity^(^ 28 ^)^.

As this is an intervention research, the results were discussed with the participants as they were produced. For the organization of the empirical material, the work of transcription was carried out, followed by the transposition, by means of a more attentive and reflexive reading, considering the speeches and the acts / gestures in the different encounters and returning them to the teams. Finally, the work of reconstitution was carried out, in which the construction of a narrative argued around the analysis of the categories^(^ 29 ^)^.

At the reconstitution stage, we sought to identify tips for the experience of a Mental Health training through PEH, that is: when analyzing the transcribed material, guided by the principles of Socio-clinic, we sought to separate statements, attitudes, behaviors, actions and postures that could be tips or points of attention to any experience of PEH and, therefore, be analyzed and discussed with the health professionals who composed this intervention research.

The research was approved by the Research Ethics Committee with the opinion of CAAE No. 53029016.2.0000.5393. The aim was to guarantee the anonymity of the participants and preserve the confidentiality of the information. The speeches were identified by the letter T, referring to worker, followed by the randomly chosen Arabic numeration, and the diary of the researcher were indicated with this nomenclature, followed by the month and year.

## Results

The experience in the process of PEH for the formation in Mental Health points to some beaconing tips guided by the Institutional Socio-clinic.

The first clue is the reflection and attention to the process of making, planning and thinking the PEH, about the instituted way of organizing and planning the trainings and activities in the service, be they graduation, postgraduate or residency activities, since the first device created was the inclusion of the management, by means of the Municipal Health Department, in the process of choosing the units participating in the study, without inserting or consulting these units on their interests in participating, restricting the choice to the Municipal Health Department.

Sometimes this organization and planning become contradictory with the proposal presented by the fact that there is collective participation in decision-making processes from the beginning of intervention planning. Including only management in this process and creating a territory of doubts, fears and longings can lead to participation in the study by obligation, seeking to satisfy management, even as a means to continue being the exemplary service, which meets what is proposed or imposed. *The students come here, they start an activity here and then they leave and we have the population to answer (T16); They came from far away to take a photo of the patient and the family is in expectation* [...]*; comes another student, comes another, at some point, it annoys and does not solve (T20).*

These reports provided a reflective look to the health team, shifting from the place already known: the place of teacher. Often, as a teacher, he enters the field of practice, enters the space and territory of the user / family and health team, and then through an administrative reorganization in the distribution of services to universities, it is necessary to change and start the practice in another unit, sometimes performing an irresponsible practice regarding the continuity of care for the other and co-responsibility in this process.

The second clue to PEH is to lose the illusion that there is control and that this is part of the movement to learn concepts, practices and to think collectively. Allowing not to control the learning process is also an important clue to self-management-based training for the collective. *What if it does not work out? What if the team does not want to? And if they do not accept it? What if they do not identify with the proposal? Will I account for this process? (Researcher’s Journal February /2016)*

In this sense, sharing the findings with the participants in a formative perspective centered on the PEH, using the restitution based on the principles of the Socio-clinic, is the third clue presented. The restitution can be an opportunity to pause to look at the process.

The purpose of the restitution was not to pass on information, but to share perceptions with the team, putting them in the analysis. The exercise was to approximate processes of self-management and self-analysis. *But how is this* [restitution]*? Are you going to tell us what you’ve written down and what you’re thinking?* (T16)*; When students come here, they record, talk, ask, and then walk away; We do not even know anything* (T20); *It was good that you talked to us about it. We already knew, but did not speak* (T19).

The fourth clue would be to look beyond what the person says in the face of situations of tension and tensions in the encounters, paying attention to what their speech carries, what “ghosts”, previous experiences, that is, the institutions that cross them. *When we began the evaluation of the meeting, I began to return to them about my perception of the group’s lack of interest in the delays, demarcations, use of cell phones, among others; naturally, statements were made that resumed restitution. Then, they began to verbalize the obligation to participate in the group (Researcher’s Diary August /2016); No one has explained anything to us until you arrive. They called here, they warned you that you would come and that it was something of mental health (T9).*

The participation of the management in the indication of the participating units brought a resistance effect to the non-explanation, consultation and discussion in team before the entrance in the field. The team has always experienced the process of heterogeneity, that is, from the control and action of others, in a relationship of vertically hierarchical power. Therefore, attention should be paid to what the speech pronounced in the meetings carries.

The implication analysis of the researcher is the fifth clue in which, through this movement, it was sometimes possible to redirect acts and behaviors as the implications were identified.

The entry into the field was initiated on the basis of a misguided and over-implied reasoning that the desire, the ideologies and implications of the researcher were in accordance with the desire, ideologies and implications of the workers and managers. A misunderstanding that may occur in other training experiences centered on the perspective of PEH, which can be redirected from the analysis of implication.

The structural-professional implication with the formation process and with the specialty in Mental Health and the existence of a psycho-affective level with the militancy in Mental Health resulted in fear and insecurity related to the fact of being a teacher of a Higher Education Institution with work in Health Mental. *I think I have demanded a lot from this intervention. Required to respond correctly in response to an expectation of the willingness to implement the Psychosocial Care Network, to walk with Psychosocial Care, to involve and bring network workers closer to mental health care (Researcher’s Journal May/2016).*

As a means of problematizing Mental Health care in the service during the PEH process, the group was offered a discussion about the work process, while a reflection of the daily practices, rethinking the object, instrument and purpose of work in Mental Health from the contributions of Mendes-Gonçalves^(^ 30 ^)^, guiding the formation in Mental Health in the context of work, provoking reflections and provocations on the daily life of these practices, being this the sixth clue for the development of PEH actions. *We did our work and did not think about the object, instrument and purpose; I had never stopped to think about it, to reflect, for a year doing my job and I did not even know what I was doing (T13); I go for the visit and often have not even stopped to think about the purpose.* [...] *now, I keep thinking about the way to the patient’s home (T8); My object of work was the disease itself, the madness, because I saw the symptom more* [...]*, now, I cannot think about it* [...] *in the subject (T3).*

And the seventh track is the indication of learning how to facilitate and experience processes of PEH in the act of doing, taking into account PEH’s own perspective that is, putting into action trying to do, in practice, in the daily work. *We were going, doing, when he realized, he was already doing Permanent Education. (T19)*

## Discussion

It is a challenge to construct and think about effective ways of training workers from their health work context so that decision-making processes are based on the collective so that workers and users actively participate^(^ 31 ^)^. Not recognizing that the organization of the health work process is a result of the inclusion and interaction of workers and users can produce bureaucratic forms of work, with the subjective impoverishment of the worker and, consequently, of the care^(^ 32 ^)^. Thus, the worker may lose his desire for work as he fails to recognize himself in the final product of his work process.

Heterogeneous management can increase the exploitation of labor, alienation, reproduction of the instituted and the naturalization of daily life, and in response to this context, self-managed proposals may arise as an institutional movement^(^ 33 ^)^.

The training process (undergraduate, graduate, residences and in-service training) is not owned by PEH. If the PEH facilitator does not take care of the construction of knowledge in the collective, taking responsibility for the process itself, it can reproduce the instituted way of functioning of the institutions, preventing the unprecedented occurrence^(^ 2 ^)^, as well as the creative and creative power of the collective.

The course of the PEH happens from the movement of the collective, the joint construction, the problematization directed to the needs. Therefore, there is no timetable or lesson plan to follow; there are agreements and directives, based on the principles of NPEP.

The restitution, as an opportunity to deepen or question the analyzes^(^ 21 ^)^, was another Socio-clinic principle used to experience PEH in the training in service in Mental Health, which also could favor the movement of the collective and the notion of lack of control.

In doing the restitution and sharing of the PEH process in Mental Health, the workers also occupied a place of actors of the learning movement, being able to speak of institutional attitudes and interferences as: to rethink and to talk about the obligation to participate and its relation with behaviors of resistance, stiffness and tension in the encounters.

Looking beyond the behaviors and speeches presented, comprising, therefore, the institutions that cross the professionals, could favor the understanding and resolution of conflicts in the process of formation. In this case, resistance as a barrier to the development of PEH should not be taken as a negative sense, but an opportunity or a way to preserve a certain way of working / life in the face of a change of course, as well as possibilities of facing some imposed determinations by management^(^ 34 ^)^.

The principles of Institutional Socio-clinic favored that the facilitator of PEH (and researcher) could interpret this stiffening in the group as a possibility of growth and involvement with in-service training, stimulating instituting processes of self-management.

Being aware of these institutional interferences, such as the fact that municipal management includes services in a taxing way, has opened the possibility for a more powerful meeting. Otherwise, disputes, disagreements and conflicts could be established^(^ 35 ^-^ 36 ^)^.

During the restitution, there was no recrimination or accusations charged with impotent denunciations, but it was used as a space for the enunciation of things, being constructive and respectful^(^ 22 ^)^.

Through the restitution, it was possible to review the demand of the group, understanding that it is changing and does not necessarily happen explicitly. The process of re-discussion of demands was relevant to the analysis of the implication of the researcher and facilitator of PEH, since, from the planning of the research as a member of the Psychiatric Reform and a specialist in Mental Health, she sought to meet a goal of transforming practices service through PEH.

Analyzing these implications with the object of study, based on Socio-clinic, favored new directions and attention for the PEH process, presenting a great contribution to the PEH facilitator in order to reflect on how the researcher relates to his object of study and work and in what form this interaction takes place - analysis of implication^(^ 25 ^)^. The implication can be divided, didactically, into three dimensions: affective-libidinal, related to the affections, of liking or not, of the empathy present or absent between the researcher and the object of study; historical-existential are the world views that carry themselves and structural-professional are the issues related to work^(^ 37 ^)^. Therefore, it was possible to go through auto-analytical processes of analysis of the implication of the researcher with his research, going through the dimensions of this implication.

This understanding of the implications allowed the researcher to understand that the need for control or destabilization against non-control, as presented previously, was related to the implication with her work as a teacher, that is, the teaching institution went through the research. Just as motivated by its ideology and affection with Mental Health care, it remained over-motivated, acting from an objective of implementing Mental Health actions in basic care in articulation with the other services of the health network.

The overlap also experienced is the result of strong ideological engagements^(^ 21 ^)^ - in this context, the militancy and specialization in Mental Health. The objective is not to make the overlap disappear, but to perceive it, to make it visible and analyzable, consequently, no longer acting in the field of overlapping, but of implication, producing a new knowledge^(^ 38 ^)^.

Making PEH for Mental Health training and being concomitantly guided by the principles of Socio-clinic favor spaces and opportunities to re-discuss and revise the needs of the group, revisit the initial proposal and understand that the PEH is a field of dialogue that should also consider the institutional interferences , the diverse implications and desires of all involved.

Another point consisted in using the concepts of object, instrument and purpose, to review the work process. The work process is a means of constructing subjectivities where the subject establishes relationships and (re)produces their existence, having as elements the object, in which the work will be applied, through the use of instruments, which are material or non-material forms (such as knowledge, individual consultation, medicine, among others) in order to achieve a specific purpose^(^ 30 ^)^.

Perceiving these aspects of the work process is also a clue so that one can progress in the transformation of professional practices from the reflective look to the daily life of these.

In this way of learning from the collective, many workers pointed to paths that widened their gaze to the person in mental distress and / or use of psychoactive substance (PAS), while reflecting on the object, instrument and purpose of their job. Thus, PEH, guided by the principles of Socio-clinic, can lead to unlearning, de-territorializations and reflections.

The movement of de-territorialization mobilized by the PEH also traverses this perception about the work, of the institutional implications, that is to say, if one intends to advance in the discussions about what has been produced of effectiveness and power in the care, and the reflection on the clinic, on the work process is relevant and can point to change paths not by the imposition of knowledge or determinations of practices, but by the joint construction of these.

Thus, as the encounters occurred, the team also began to be clear about the proposal, since it also started an active participation in the discussions, planning and evaluations of the meetings, understanding that the facilitator was not in a position to pass on information, but to construct meanings that would transform the practice of health work collectively, resulting in PEH actions.

These processes have made the facilitator / worker and the workers face difficult scenes such as: not knowing; do not control; the preconceptions; the exercise of a look at oneself and at one’s own practice; to question the reason for certain attitudes routinely committed mechanized, given as natural, generating a certain burden of suffering. In this movement, building the formation from the context, fragilities and potentialities was something powerful and transformative.

Care in Mental Health involves legal-political aspects, training, ethical and political values in which care is centered on the user / family. In considering these aspects, it is no longer possible to think about Mental Health care restricted to medicine, centered on medical knowledge and power and standardized solutions, but based on wholeness, in the context of the subject, in their psychosocial singularity.

That is, the transformation of the look into the work object leads to the creation of new instruments based on inter-sectoral actions that seek the social reinsertion and the autonomy of the users^(^ 39 ^)^. Facing this, it is noticed that the meetings of PEH favored moments of learning to learn, deconstructing and reconstructing new knowledge.

This critical capacity to look at everyday practice and activities with a willing eye to identify fragilities, but also inventions / creations, permeates the process of perceiving reality as being dynamic and fruit of a social and historical construction, which can be transformed^(^ 40 ^)^.

In this sense, PEH participants began to unveil reality in a movement to look at themselves and practice. Therefore, it consisted in thinking of the movements of PEH as a capacity for outreach, that is, of discovering / producing / welcoming others within ourselves; a multiple singularity, stimulating the capacity of creation, of unfolding, of curiosity before the world, activating the power before the life^(^ 41 ^)^.

As limit of the study, it is pointed out the non-discussion of results with municipal management. It is considered that the qualitative research does not have the ambition of generalization of results but of the reflections. In this way, it is possible that PEH takes place from the reality of each day-to-day life, so it is not proposed (and should not be), with this research, to construct a training manual based on the principles and guidelines of the PEH, but point out tips that facilitate its development in the act.

## Conclusion

PEH can be a practice that mobilizes de-territorializations in health work teams as it produces reflection and self-analysis. But it can also be captured by instituted models and by specialized looks for Mental Health as specificity, leading to a more objective, positivist formation with few flexibilities and possibilities of speech or creation in the group and by the group. Thus, it is considered that Institutional Socio-clinic, as a beacon of a process of Mental Health training through PEH, can favor or facilitate movements of looking and perceiving these captures of the instituting forces in daily life.

The Institutional Socio-clinic can potentiate the PEH process, pointing out tips to the formation process that can also be strategies for the production of knowledge in the work and for strengthening the inclusion of the subjects in the collective construction of learning.

It is emphasized that the seven tips presented are not configured as protocols, but are designed to assist facilitators and workers involved in the PEH process. Regardless of the purpose of the training - in this study, the focus is on Mental Health -, the tips are indications to consider the crossings and institutional interferences in health care and, consequently, for training and for the work.
